# Clinical Identification and Characteristic Analysis of Giant Cell Myocarditis in 12 Cases

**DOI:** 10.3389/fcvm.2021.649094

**Published:** 2021-04-13

**Authors:** Shangyu Liu, Lihui Zheng, Lishui Shen, Lingmin Wu, Yan Yao

**Affiliations:** State Key Laboratory of Cardiovascular Disease, National Center for Cardiovascular Diseases, Fuwai Hospital, Chinese Academy of Medical Sciences and Peking Union Medical College, Beijing, China

**Keywords:** giant cell myocarditis, ventricular tachycardia, arrhythmogenic right ventricular cardiomyopathy, endocardial myocardial biopsy, pathology

## Abstract

**Aims:** Giant cell myocarditis (GCM) is a rare, rapidly progressing cardiomyopathy with high mortality, if not diagnosed and treated in time. We analyzed the progression and clinical manifestations of patients with definitive diagnosis of GCM.

**Methods and Result:** We enrolled 12 patients diagnosed with GCM in the explanted heart during heart transplantation (HTx) or by endomyocardial biopsy (EMB) and collected information on demographic data, cardiac structure and function, arrhythmias, preliminary diagnosis, and delay of the diagnosis. Seven cases were diagnosed from biopsy samples during HTx, and five cases were diagnosed through EMB. Before the diagnosis of GCM based on pathological analysis, these patients had been incorrectly diagnosed with arrhythmogenic right ventricular cardiomyopathy (*n* = 5), dilated cardiomyopathy (*n* = 2), ventricular tachycardia (*n* = 2), viral myocarditis (*n* = 1), cardiac amyloidosis (*n* = 1), and ischemic cardiomyopathy (*n* = 1) based on clues such as symptoms, arrhythmia, and cardiac imaging. Patients diagnosed with GCM through EMB had a shorter symptom-onset-to-diagnosis time (6.6 ± 2.7 months) and milder heart damage (left ventricular ejection fraction, 47.2 ± 8.8%) than those diagnosed during HTx (11.0 ± 3.3 months, *P* = 0.034; 31.4 ± 10.9%, *P* = 0.024).

**Conclusion:** GCM is easily misdiagnosed as other types of myocarditis and cardiomyopathy. Pathological examination of the myocardium is the most reliable diagnostic method for GCM. Endocardial biopsy can identify patients with GCM at an earlier stage.

## What's New?

- Giant cell myocarditis (GCM) combined with cardiac arrhythmias such as heart block and ventricular tachycardia, impaired cardiac structure, and function is often misdiagnosed as other types of myocarditis or cardiomyopathy.
- The typical manifestation in the histopathological examination of the myocardium is the most effective diagnosis method of GCM.
- Endocardial myocardial biopsy can identify patients with GCM in earlier stages when the cardiac function is less impaired.

## Introduction

Giant cell myocarditis (GCM) is a rare and highly fatal type of cardiomyopathy (1). Myocardial cell necrosis, multifocal or diffuse inflammatory cell infiltration, and characteristic multinucleated giant cells are typical pathological manifestations of GCM (2). Due to the rapid progression of GCM, early and accurate identification and timely initiation of immunosuppressive therapy can significantly improve the prognosis (1, 3). GCM patients often seek medical attention for progressive heart failure and arrhythmia, with atypical symptoms and clinical manifestations. In some case reports, patients have experienced a long process of misdiagnosis and mistreatment, and by the time GCM is diagnosed histopathologically, they have lost the best window for treatment (4, 5). Therefore, we aimed to analyse the process, progression, and clinical manifestations of patients with a definitive diagnosis of GCM supported by myocardial pathologic examination to help identify this disease in a more timely and effective manner.

## Methods

### Study Population

From June 2015 to April 2020, 12 consecutive patients diagnosed with GCM at Fuwai Hospital were enrolled in the study. The heart samples were taken from the recipients of heart transplantation (HTx) or endomyocardial biopsy (EMB). Hematoxylin-eosin (H&E) staining was used to determine myocyte degradation and inflammatory-cell infiltration and fibrosis in the full-thickness myocardial sections or multi-site biopsy. All pathologies were diagnosed. For each patient, the following information was collected from the medical records: clinical manifestation, cardiac imaging examinations performed by echocardiography, cardiac magnetic resonance, emission computed tomography, and electrocardiograms (ECGs).

The diagnosis of GCM mainly relied on clear pathology based on typical lesions, including multinucleated giant cells accompanied by numerous inflammatory immune cells. The normal myocardium was diminished and replaced by fibrous tissues containing scattered multinucleated giant cells. The data on the patient's previous diagnosis based on medical history, clinical manifestations, and other examinations were also collected. The diagnosis of arrhythmogenic right ventricular cardiomyopathy (ARVC) was established based on the 2010 Task Force Criteria (6). The diagnosis of dilated cardiomyopathy (DCM) was based on the current guidelines as well as scientific statements regarding cardiomyopathy by experienced cardiologists (7, 8). Idiopathic ventricular tachycardia (IVT) was diagnosed based on the duration of monomorphic ventricular tachycardia ≥30 s or <30 s. However, the onset of ventricular tachycardia with hemodynamic disorders required early intervention, and no evidence of structural heart disease was found (9–11). The diagnostic criteria for viral myocarditis were referred to the 2013 European Society of Cardiology's Consensus on the etiology, diagnosis, management, and treatment of myocarditis (12). The diagnosis of myocardial amyloidosis was based on its imaging features with consensus (13). Ischemic cardiomyopathy was based on the patient's history of myocardial ischemia. This study was conducted per the Declaration of Helsinki and was approved by the Institutional Ethical Committee.

### Statistical Analyses

Statistical analyses were performed using the IBM SPSS Statistics for Windows, version 20 (IBM Corp., Armonk, NY). Continuous variables are presented as mean ± standard deviation and categorical variables as the number of cases and percentage. The Student's *t*-test for continuous variables with correction for unequal variance was used when necessary. *P* < 0.05 were considered statistically significant.

## Results

### Demographic and Clinical Characteristics of GCM

The patient demographic and clinical characteristics are shown in Table 1. From a total of 12 enrolled patients, five cases were diagnosed through EMB, and seven cases were diagnosed by the recipient's biopsy cardiac pathological examination at the time of HTx. Among all GCM patients, six were male (50%), the average age was 46.1 ± 9.6 years, the maximum age was 55 years, and the minimum was 20 years. The average left ventricular ejection fraction (LVEF) was 38.0 ± 12.6%. Except for two patients—one with Hashimoto's thyroiditis and the other with orbital inflammatory pseudotumor—the remaining patients had no autoimmune diseases. Six patients had already undergone radiofrequency ablation for ventricular arrhythmia before our diagnosis.

**Table 1:** Patient characteristics.

**Patients**	**Age**	**Sex**	**LVEF (%)**	**Autoimmune**	**Radiofrequency**	**Sample**
	**(years)**			**diseases**	**ablation**	**source**
#1	45	M	31	None	No	HTx
#2	47	M	35	None	No	HTx
#3	34	M	20	None	No	HTx
#4	52	F	39	None	Yes	HTx
#5	53	F	20	None	No	HTx
#6	51	F	50	None	No	HTx
#7	55	F	25	None	No	HTx
#8	52	M	43	HT	Yes	EMB
#9	21	F	35	OIPT	Yes	EMB
#10	50	M	58	None	Yes	EMB
#11	46	F	52	None	Yes	EMB
#12	47	M	48	None	Yes	EMB

*M, Male; F, Female; HT, Hashimoto's thyroiditis; OIPT, Orbital inflammatory pseudotumor; HTx, Heart transplantation; EMB, Endomyocardial biopsy*.

In the subgroup comparison, patients with HTx were aged 48.1 ± 7.1 years and had an LVEF of 31.4 ± 10.9% whereas patients with EMB were aged 43.20 ± 12.6 years and had an LVEF of 47.2 ± 8.8%. No significant difference in age was found between the two groups (*P* = 0.405); however, the patients in the EMB group had better indicators of the heart function (LVEF) compared to HTx patients (*P* = 0.024).

We found 11 (91.7%) patients with arrhythmia, among which there were 9 (75.0%) patients with different degrees of atrioventricular block and/or right bundle branch block (RBBB), 10 (83.3%) patients with ventricular tachycardia, and 1 (8.3%) patient with atrial tachycardia. There were 8 (66.7%) patients who simultaneously developed heart block and ventricular tachycardia.

All patients underwent more than one type of cardiac imaging (echocardiography, cardiac magnetic resonance, and emission computed tomography). The examinations showed no characteristic features of GCM; it manifested as expansion or contraction of a single ventricle, double ventricle dysfunction, or thickened interventricular septum (Table 2).

**Table 2:** Arrhythmia, imaging results, and diagnosis process.

**Patients**	**Bradycardia/heart block**	**Ventricular arrhythmia**	**Atrial arrhythmia**	**Medical imaging**	**Previous diagnosis**	**Time from symptoms to diagnosis (months)**
#1	None	None	None	Echo, Wholeheartedly expand. CMR: Wholeheartedly expand	DCM	15
#2	I°AVB, RBBB	VT	None	Echo: Wholeheartedly expand	DCM	12
#3	III°AVB, RBBB	VT	None	Echo: Wholeheartedly expand. CMR: Wholeheartedly expand, right ventricular wall fat replacement	ARVC	9
#4	None	VT	None	Echo: Biventricular enlargement, thickened interventricular septum. CMR: Wholeheartedly expand, right ventricular wall fat replacement	ARVC	10
#5	III°AVB	None	None	Echo: Weakened ventricular movement	ICM	6
#6	III°AVB	VT	AT	Echo: Wholeheartedly expand. CMR: Wholeheartedly expand, multiple delay enhancements	ARVC	15
#7	None	VT	None	Echo: Dilated right ventricle, thinned free wall of right ventricle	ARVC	10
#8	I°AVB,RBBB	VT	None	Echo: Right ventricular dilation and septal thickening	IVT	11
#9	RBBB	VT	None	Echo: Biventricular dilation, contractile dysfunction	ARVC	5
#10	RBBB	VT	None	Echo: No obvious abnormalities. CMR: No obvious abnormalities	IVT	7
#11	RBBB	VT	None	Echo: Ventricular septum and left ventricular wall thickening. CMR: The left ventricular wall thickens and the ventricular septal limitation strengthens. ECT: Increased uneven metabolism of the right ventricular wall	Amyloidosis	6
#12	RBBB	VT	None	Echo: Enlarged left ventricle. CMR: Enlarged left ventricle, delayed enhancement of left ventricle and septum	Viral myocarditis	4

*AVB, Atrioventricular block; RBBB, Right bundle branch block; VT, Ventricular tachycardia; AT, Atrial tachycardia; Echo, Echocardiography; CMR, Cardiac magnetic resonance; ECT, Emission computed tomography; DCM, dilated cardiomyopathy; ARVC, arrhythmogenic right ventricular cardiomyopathy; ICM, ischemic cardiomyopathy; IVT, Idiopathic ventricular tachycardia*.

### GCM Diagnosis Process

**Figure 1:**
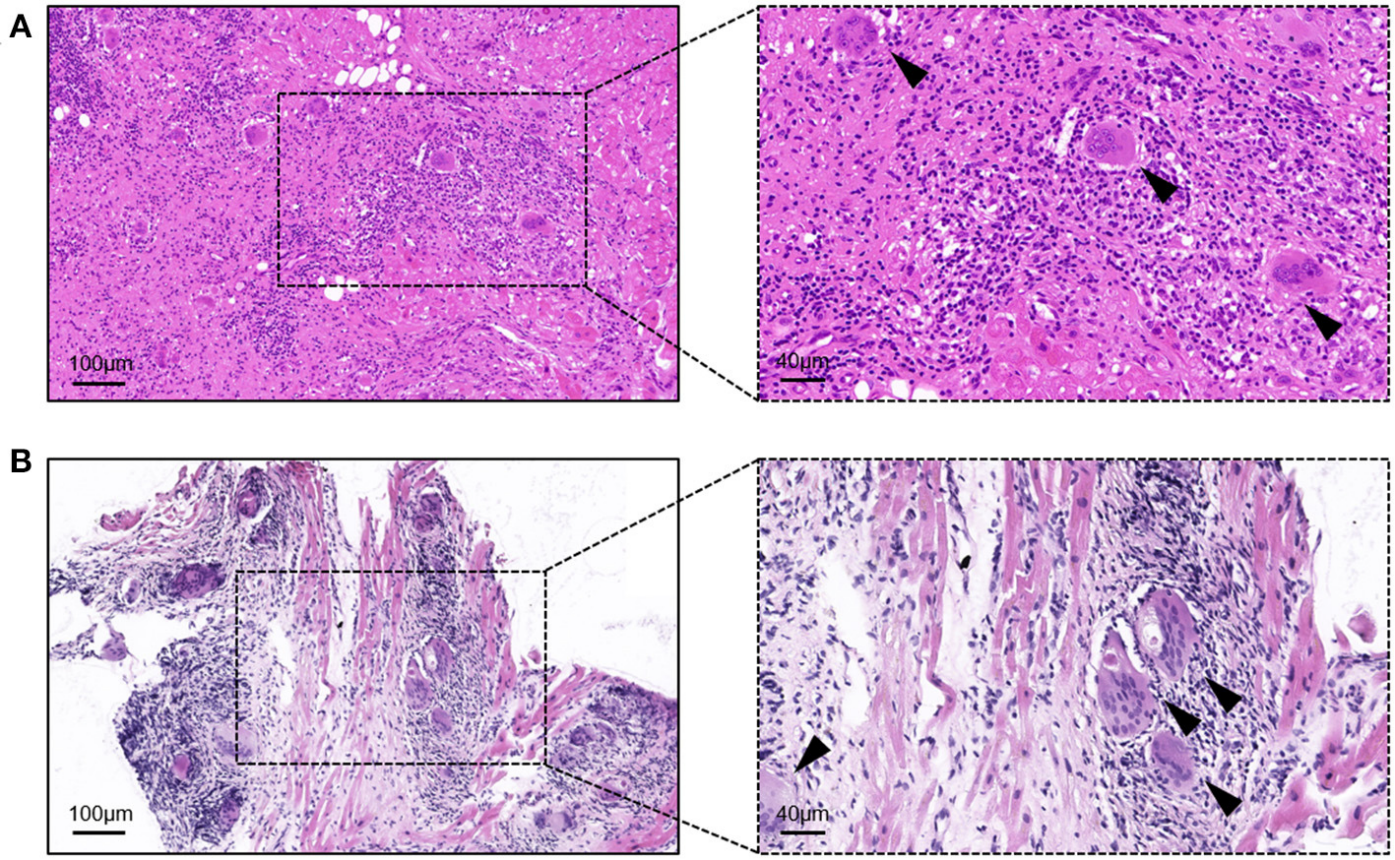
Hematoxylin-eosin staining of the samples from giant cell myocarditis patients. **(A)** Sample from the heart transplantation; and **(B)** sample from the endomyocardial biopsy. Arrows indicate typical multinucleated giant cells.

Before starting the initial treatment, all patients had not completed the pathological diagnosis. Regardless of the method of obtaining the samples (i.e., HTx or EMB), the pathological performance was consistent with the typical characteristics of GCM. The typical GCM histopathological feature is myocardial cell necrosis accompanied by multifocal or diffuse inflammatory cell infiltration, multinucleated giant cells, eosinophils, and neutrophils (Figure 1).

**Figure 2:**
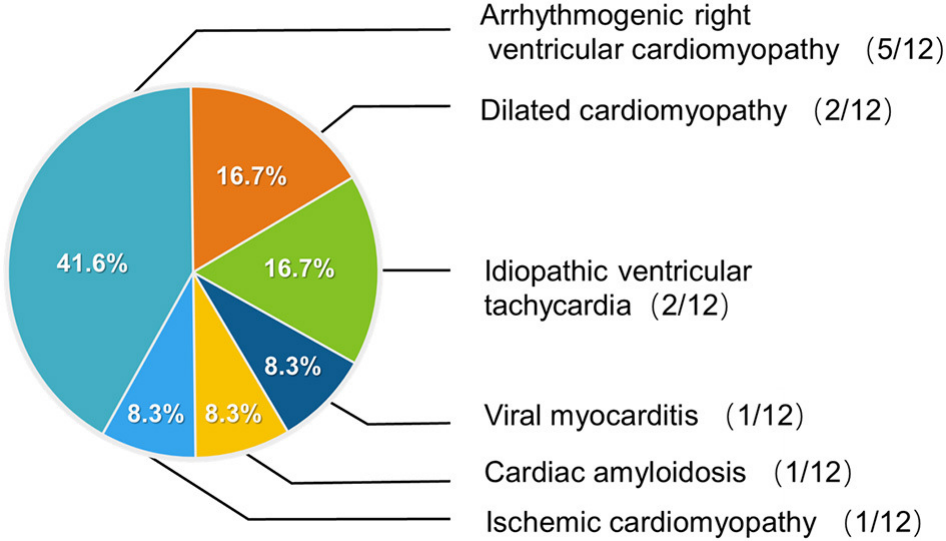
Previous diagnosis of patients with giant cell myocarditis.

Based on case history, clinical manifestations, and other examinations, the data on the previous diagnosis was collected for all patients. Among them, 5 (41.6%) patients were diagnosed with arrhythmogenic right ventricular cardiomyopathy (ARVC), 2 (16.7%) with dilated cardiomyopathy (DCM), 2 (16.7%) with idiopathic ventricular tachycardia (IVT), 1 (8.3%) with viral myocarditis, 1 (8.3%) with cardiac amyloidosis, and 1 (8.3%) with ischemic cardiomyopathy due to his history of the disease (Figure 2). We re-examined the basis of the diagnosis at the time. In the absence of myocardial biopsy and based on the patient's symptoms, arrhythmia, along with cardiac structure and function, these diagnoses were plausible.

**Figure 3:**
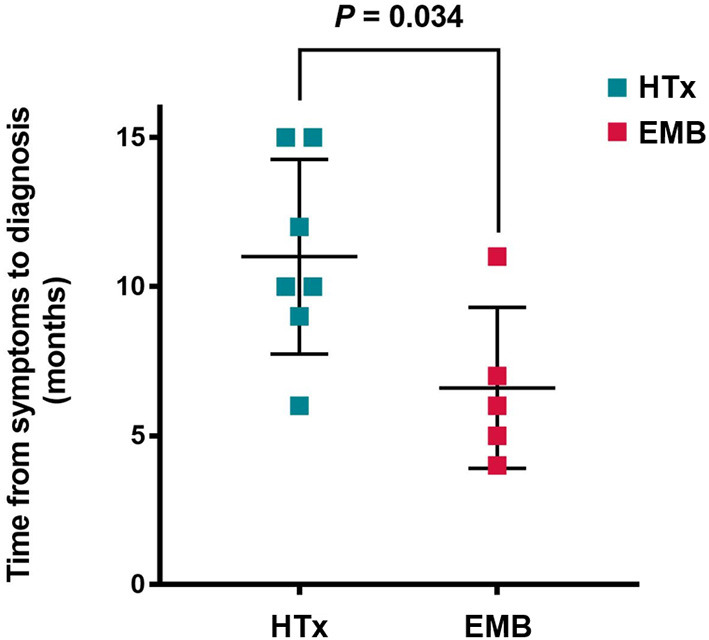
Time from symptom onset to diagnosis of giant cell myocarditis. HTx, Heart transplantation; EMB, Endomyocardial biopsy.

Before obtaining a definite diagnosis, all patients had not received special treatment for GCM. The average delay was 9.2 ± 3.7 months, from the occurrence of GCM-related symptoms to the pathological examination, wherein the longest period was 15 months and the shortest was 4 months. Based on the comparison between the subgroups of HTx and EMB, the definite delay time was 11.0 ± 3.3 months for the HTx patients and 6.6 ± 2.7 months for the EMB patients. The time required for the diagnosis of GCM for the patients with EMB was significantly shorter than that for the HTx patients (*P* = 0.034, Figure 3).

## Discussion

We found that all the included GCM patients had a definite diagnosis through pathology. Prior to this, they had been initially misdiagnosed with other types of myocarditis or cardiomyopathy based on arrhythmia or cardiac imaging and did not receive targeted treatment in time. Patients with GCM diagnosed through EMB had a shorter delay from the onset of symptoms to diagnosis compared to the patients with HTx and a relatively better heart function.

Most of the patients in our research had a heart block of varying degree, bradyarrhythmia such as RBBB and ventricular tachycardia, while one patient had atrial arrhythmia. Cardiac imaging generally showed dilation of the heart and thickening of the ventricular septum, but it did not display any characteristic features. GCM can appear as an enlarged heart and ventricular tachycardia in its early stages, and individual patients can also have epsilon waves in the ECG, thereby meeting the key diagnostic criteria of ARVC and leading to misdiagnosis (6, 14). In our study, 5 out of 12 GCM patients had been misdiagnosed with ARVC. Previous studies have shown a relationship between GCM and autoimmune diseases (6, 15–17). One of the 12 patients we included had Hashimoto's thyroiditis and another had orbital inflammatory pseudotumor, proving the high level of suspicion of GCM. In addition, GCM, ARVC, sarcoidosis, cardiac amyloidosis, other cardiomyopathies, and myocarditis are rare diseases. Doctors' lack of knowledge about the diagnosis and differential diagnosis of these diseases is also one of the reasons for misdiagnosis.

It is undeniable that we currently have no evidence that these patients have GCM combined with cardiomyopathy such as ARVC. However, it is worth noting that the most significant difference between treatments for GCM and cardiomyopathies, such as in DCM and ARVC, treatment is that the former requires the use of immunosuppressive agents, meaning that early recognition of GCM and timely targeted treatment can provide the most benefit. Studies have shown that the use of immunosuppressive agents, such as prednisone, cyclosporine, and azathioprine, could extend the average survival time of the patients with GCM from 3 months to 1 year (1). Combining new immunosuppressive drugs has also achieved excellent results in the treatment of GCM (3). According to some autopsy reports, some patients do not receive timely and effective treatment, leading to rapidly deteriorating heart function and sudden cardiac death (18–20). Heart transplantation is preserved as the treatment only for end-stage GCM patients due to the limited source of donors, and this is currently not a common treatment. Concurrently, treatments of heart failure and arrhythmia are essential. In our study, most patients with ventricular tachycardia received radiofrequency ablation. It has been reported that percutaneous mechanical circulatory support can also be used for GCM patients with severe heart failure (21).

Compared with patients diagnosed by EMB, the cardiac function of patients who had heart transplantation was lower. We assumed that this may be due to the misdiagnosis or delayed diagnosis of GCM. One of the most significant clinical manifestations of patients with GCM is progressive systolic heart failure. Poor cardiac function with arrhythmia may conceal the real cause and delay targeted treatment. Therefore, early detection and verification through pathological tests are particularly important in ruling out GCM.

Histopathological examination is the “gold standard” for diagnosing myocarditis and cardiomyopathy, by identifying the disorder and hypertrophy of myocardial cells, fibrous tissue hyperplasia, fat cell replacement, and infiltration of inflammatory cells (22). Congo red staining can be used to identify the amyloid and several other immunohistochemical staining techniques could be used to distinguishes different types of immunoinflammatory cells (23, 24). However, awareness regarding the importance of histopathological examination in the diagnosis of myocarditis and cardiomyopathy remains low. Most of the histopathological examinations are performed either on the recipient's heart or during an autopsy after the heart transplantation, and rarely *in vivo*. Based on several autopsy reports, the incidence of GCM has been reported to range from 0.007 to 0.051%; however, this number might be underreported as an autopsy is not routinely performed for all unexplained cases and sudden cardiac deaths (2, 25, 26).

The biopsy of myocardial intima is the most suitable method for examination in the early stage. Since its introduction into clinical practice in 1963 by Sekiguchi et al. (27), endomyocardial biopsy has gradually become an important clinical procedure with great diagnostic value. According to a scientific statement from the American College of Cardiology/American Heart Association and the European Society of Cardiology, patients with unexplained heart failure symptom duration of shorter than 2 weeks and a normal or enlarged left ventricle accompanied by hemodynamic disorders should undergo endomyocardial biopsy (Class IB) (28). However, it is frustrating that a vast majority of medical institutions, including our center, lack sufficient knowledge of the diagnostic value of EMB in myocarditis and cardiomyopathy, owing to which many patients fail to undergo myocardial histopathology in time. Also, the implementation rate of EMB is also much lower than the actual demand. Although the safety of EMB procedures has been confirmed, more accurate targeting of high-risk patients will contribute to the adoption of EMB examination (29, 30). The diagnosis of cardiomyopathy, such as ARVC, is highly suspected, especially combined with ventricular tachycardia and heart block, should also be considered to determine the true cause. In our study, patients diagnosed with GCM through EMB had a shorter time from disease-related symptom onset to diagnosis compared to patients with HTx. They also had a better cardiac function, which offered sufficient time for targeted treatment. Combined with autoimmune diseases, the presence of tachyarrhythmia and bradyarrhythmia, with or without cardiac structural and functional abnormalities, may be potential criteria to prompt clearly or exclude GCM through EMB as soon as possible to develop more targeted strategies without delaying treatment. Based on the current limited knowledge about GCM, effective identification of GCM will require a joint effort from cardiologists.

### Limitations

Due to the circumstances of this study, we could only analyze the patients diagnosed with GCM through histopathological examination. More GCM patients are expected with no definite diagnosis either due to the absence of EMB or other cardiac pathological examinations. Because some patients undergo HTx and require immunosuppressive agents to avoid rejection, only patients diagnosed through EMB received immunosuppressive therapy. Furthermore, we did not analyse the prognosis of patients.

## Conclusions

In conclusion, most patients with GCM have concomitant heart block and ventricular tachycardia and damaged cardiac structure and function. Before undergoing a histopathological examination, the patients are often misdiagnosed with ARVC or other cardiomyopathies. EMB is an effective method to detect GCM. Patients diagnosed with GCM through EMB have a shorter period from symptom onset to diagnosis and milder heart damage improving the overall outcome.

## Data Availability Statement

The original contributions generated for the study are included in the article/Supplementary Material, further inquiries can be directed to the corresponding author/s.

## Ethics Statement

Written informed consent was obtained from the individual(s) for the publication of any potentially identifiable images or data included in this article.

## Author Contributions

SL was the main finishers of this research development, data collection, and analysis. LZ and LS were responsible for data collection. LW was responsible for the verification of research pathology results. As the corresponding authors of this article, YY reviewed all the results of the article and critically revised the manuscript. All authors have contributed substantially to the completion of this research.

## Conflict of Interest

The authors declare that the research was conducted in the absence of any commercial or financial relationships that could be construed as a potential conflict of interest.
