# Supplementary material for: Clinical Identification and Characteristic Analysis of Giant Cell Myocarditis in 12 Cases
Source: Front Cardiovasc Med. 2021 Apr 13;8:649094. doi: 10.3389/fcvm.2021.649094 (PMC8076517; doi:10.3389/fcvm.2021.649094)
Supplement: Supplementary file 1 [file Data_Sheet_1.docx]

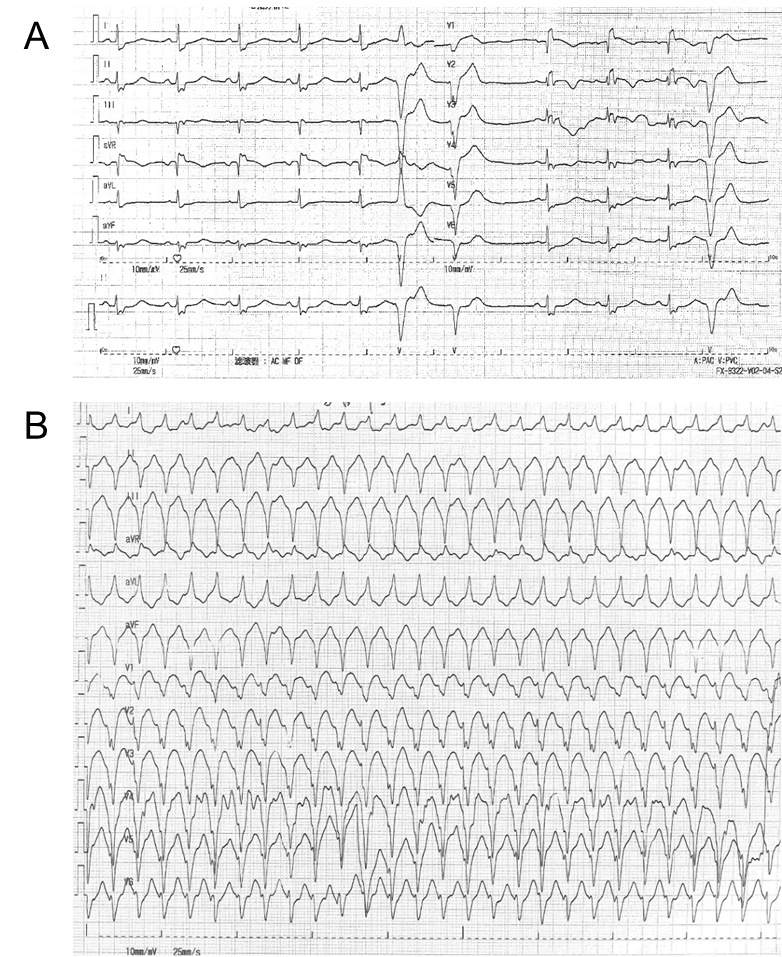


**Supplementary Data 1** Electrocardiogram performance of giant cell myocarditis patients. (A) Complete right bundle branch block; and (B) ventricular tachycardia.


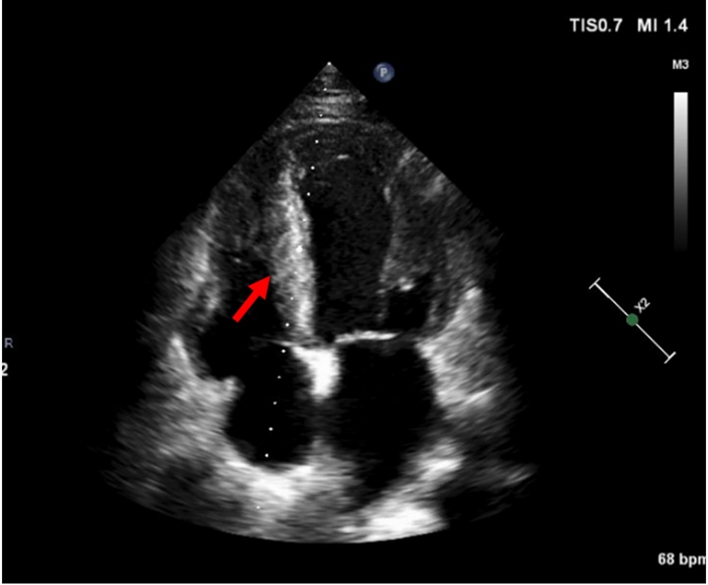


**Supplementary Data 2** Echocardiography in patients with giant cell myocarditis (prompts thickened ventricular septum). Arrows indicate thickened ventricular septum.

**Supplementary Data 3** Dosing of the key immunosuppressive drugs in patients undergoing endocardial biopsy

|  | Patients | | | | |
| --- | --- | --- | --- | --- | --- |
| Patients | #8 | #9 | #10 | #11 | #12 |
| Prednisone, mg | 50 | 50 | 60 | 50 | 50 |
| Mycophenolate mofetil, g | 1 | 1 | 1 | 1 | 1 |
| Cyclosporine, mg | 150 | 150 | 175 | 150 | 150 |

The data is based on the daily dosage of the patient at the initial stage.
